# Mechanism-Based Urinary Biomarkers to Identify the Potential for Aminoglycoside-Induced Nephrotoxicity in Premature Neonates: A Proof-of-Concept Study

**DOI:** 10.1371/journal.pone.0043809

**Published:** 2012-08-24

**Authors:** Stephen J. McWilliam, Daniel J. Antoine, Venkata Sabbisetti, Mark A. Turner, Tracey Farragher, Joseph V. Bonventre, B. Kevin Park, Rosalind L. Smyth, Munir Pirmohamed

**Affiliations:** 1 MRC Centre for Drug Safety Science, Department of Molecular and Clinical Pharmacology, University of Liverpool, Liverpool, United Kingdom; 2 Department of Medicine, Renal Division, Brigham and Women’s Hospital, Harvard Medical School, Boston, Massachusetts, United States of America; 3 Department of Women’s and Children’s Health, Neonatal Unit, Liverpool Women’s Hospital, University of Liverpool, Liverpool, United Kingdom; 4 Leeds Institute of Health Sciences, University of Leeds, Leeds, United Kingdom; 5 Institute of Child Health, Department of Women’s and Children’s Health, University of Liverpool, Liverpool, United Kingdom; University of Sao Paulo Medical School, Brazil

## Abstract

Premature infants are frequently exposed to aminoglycoside antibiotics. Novel urinary biomarkers may provide a non-invasive means for the early identification of aminoglycoside-related proximal tubule renal toxicity, to enable adjustment of treatment and identification of infants at risk of long-term renal impairment. In this proof-of-concept study, urine samples were collected from 41 premature neonates (≤32 weeks gestation) at least once per week, and daily during courses of gentamicin, and for 3 days afterwards. Significant increases were observed in the three urinary biomarkers measured (Kidney Injury Molecule-1 (KIM-1), Neutrophil Gelatinase-associated Lipocalin (NGAL), and N-acetyl-β-D-glucosaminidase (NAG)) during treatment with multiple courses of gentamicin. When adjusted for potential confounders, the treatment effect of gentamicin remained significant only for KIM-1 (mean difference from not treated, 1.35 ng/mg urinary creatinine; 95% CI 0.05–2.65). Our study shows that (a) it is possible to collect serial urine samples from premature neonates, and that (b) proximal tubule specific urinary biomarkers can act as indicators of aminoglycoside-associated nephrotoxicity in this age group. Further studies to investigate the clinical utility of novel urinary biomarkers in comparison to serum creatinine need to be undertaken.

## Introduction

Acute Kidney Injury (AKI) is common in neonatal intensive care units (estimated incidence 6–24%). [1] Neonates who have AKI are at risk of later developing chronic renal disease and hypertension. [2] Premature infants are commonly exposed to potentially nephrotoxic medications (particularly aminoglycosides (AGs) and non-steroidal anti-inflammatory drugs (NSAIDs)).

AG antibiotics (commonly gentamicin) are often used, in combination with beta-lactams, for the treatment of neonatal sepsis. There is an 8 to 30% incidence of nephrotoxicity (absolute increase in serum creatinine level of 0.5 mg/dl) associated with AG exposure in older age-groups, [3] but there is a paucity of data quantifying AG-induced nephrotoxicity in neonates. [4] A study of gentamicin toxicity in rats suggested that nephrotoxicity is greatest, and occurs earlier, in the most immature animals. [5] Current strategies for the prevention of AG-induced nephrotoxicity include extended-interval dosing, and drug trough level monitoring with dose adjustment.

Accumulation of aminoglycosides within proximal tubule epithelial cells (PTECs) in the renal cortex, following glomerular filtration, by endocytosis via the multi-ligand receptor, megalin, [6] is thought to be the key determining mechanism for the development of toxicity. [7] Intracellular AG can result in apoptosis or necrosis of PTECs by a variety of pathways (mitochondrial dysfunction and the release of reactive oxygen species) [8], [9].

The traditional indicator of AKI is a rise in serum creatinine concentration, but this has a delayed response with levels rising significantly above baseline levels only when 25–50% of renal function has been lost. [2] Furthermore, it is a marker of glomerular filtration and not an indicator of proximal tubule function. Oliguria is, at best, a late sign of AKI, and may not be present in many forms of AKI especially those related to toxins. [10] Therefore, to identify infants early that are at increased risk of renal impairment there is a need for biomarkers that reflect different regions of the kidney that can be quantified earlier than currently used indicators. This would, in turn, allow for treatment adjustment and the avoidance of further injury.

A number of urinary biomarkers have been identified and have been qualified for use in pre-clinical models that hold the potential translational utility to identify drug-induced AKI in premature neonates. For example, Kidney Injury Molecule-1 (KIM-1), Neutrophil Gelatinase-associated Lipocalin (NGAL) and N-acetyl-β-D-glucosaminidase (NAG) are urine-based indicators of PTEC damage that are detectable earlier than currently used clinical standards (serum creatinine and blood urea nitrogen). Reports suggest that both NGAL and NAG hold translational application to report tubular injury in both adults [11] and children.[12]–[14] However, there is currently limited data investigating the potential utility of novel renal biomarkers to report renal injury in the neonatal population. In particular, with respect to KIM-1, prior to this study, there are no published data investigating its use for the detection of nephrotoxicity associated with AG therapy in neonates.

Therefore, the aims of this proof-of-concept study were to (a) assess the feasibility of collecting serial urine samples from premature neonates in the ICU setting; (b) measure a panel of urinary biomarkers in the small volume of samples that could be collected; and (c) determine whether elevations in these biomarkers were associated with the use of nephrotoxic therapeutics in this patient population and relate this to serum creatinine and aminoglycoside levels. We hypothesized that due to the reported utility of these biomarkers from preclinical data, elevations in all 3 biomarkers would be detected in the absence of serum creatinine changes, and in confirmed cases of AKI, elevations in these biomarkers will precede changes in serum creatinine concentration.

## Methods

### Patient Recruitment and Sample Collection

We recruited consecutive neonates with a gestational age less than or equal to 32 weeks admitted to the Neonatal Intensive Care Unit (NICU) at Liverpool Women’s Hospital, UK, between 1^st^ September 2009 and 24^th^ December 2009. Neonates were excluded if they had a known or suspected renal or chromosomal abnormality, or if they were expected to die within 48 hours of recruitment. The parents or guardians were approached between days 4 to 7 of life of each eligible neonate and provided information about the study. Informed consent was sought over the next 24 hours. The study was conducted with the approval of The Liverpool Paediatric Research Ethics Committee, University of Liverpool, UK. Informed written consent was obtained from carers or guardians on the behalf of the minors/children participants involved in our study.

An initial urine sample was collected on day 5 to 8 of life, after consent had been given. Further routine urine samples were collected at weekly intervals for the duration of their stay at the intensive care unit. Urine samples were collected as soon as possible after beginning treatment with gentamicin (usually the following morning). Samples were collected on a daily basis for the duration of the antibiotic course, and until three days after the last dose of gentamicin. Urine samples were collected using cotton wool balls placed in the perineum inside the nappy. Urine from non-faecal contaminated samples was transferred to a sterile container and then centrifuged at 2000 g for 4 min. Supernatant was then stored at −80°C.

Serum creatinine concentrations were measured (using the compensated kinetic Jaffe reaction) as part of normal clinical care for each neonate. No additional blood samples were taken, and no extra tests ordered as part of the study.

Neonates who were treated with gentamicin received a dose of 4.5 mg/kg every 36 hours in accordance with local protocols. Trough serum concentrations were measured just prior to the second dose, and then every 3 to 4 days.

The local guideline for indomethacin recommended its use as prophylaxis for intraventricular haemorrhage (IVH) in neonates with a gestational age of <28 weeks and/or birthweight <1000 g: 3 doses of 100 µg/kg, given soon after birth, and then every 24 hours. Indomethacin was also recommended for treatment of symptomatic patent ductus arteriosus (PDA) at a dose of 100 µg/kg daily for 6 days.

Given that changes in hemodynamics can result in changes in serum creatinine independent of any damage to the nephron, we have defined AKI in this study as a serum creatinine concentration >132.6micromol/l (1.5 mg/dl) which is slightly more than double the median baseline serum creatinine value and/or oliguria (urine output <1 ml/kg/hour recorded at any point). This cut-off value of serum creatinine for the definition of AKI in neonates has been commonly used in previous studies [15]. Although newer proposed criteria for the definition of AKI in neonates, such as the modified Acute Kidney Injury Network (AKIN) criteria, may be more sensitive [16], for the purposes of this study, we were most interested in identifying those neonates with the most severe AKI.

Neonates were followed-up for the duration of their admission on the unit, or until the end of the study period. Some neonates were discharged home directly from the study unit, whereas others were transferred to satellite neonatal units for further care before discharge.

### Determination of Urinary Biomarkers

Collected urine samples were thawed, mixed and centrifuged (3000 rpm, 5 min). Biomarker measurements were performed on the resulting supernatants. Urinary KIM-1 and NGAL measurements were performed using microsphere-based Luminex technology, as previously described, using 25 µl of urine per biomarker analysis, and analysed in duplicate. [17] Respective analytes were quantified using a 13-point five parametric logarithmic standard curve for each biomarker. The inter-and intra-assay variability was less than 15% for all assays. Urinary NAG was determined spectrophotometically according to the manufacturers’ protocols (Roche Diagnostics), using 5 µl of urine per analysis, and analysed in duplicate. The urinary levels of the respective analytes are expressed as absolute (KIM-1 and NGAL) and enzymic activity (NAG) values and normalized to urinary creatinine concentration. Urinary creatinine (uCr) was determined spectrophotometically as previously described, [18] using 25 µl of urine per analysis, and analysed in duplicate. Investigators performed all analyte measurements blindly and were unaware of the patients’ clinical characteristics.

### Statistical Analysis

We designed the study to combine weekly baseline measurements with more frequent, daily, measurements in neonates on gentamicin. This allowed us to determine baseline values taking account of postmenstrual age and to assess changes in these biomarkers. We hypothesised that we would see an increase in each marker related to gentamicin administration due to PTEC damage.

To assess the association between each biomarker and gentamicin treatment, two aspects of the change in each biomarker over the follow-up must first be accounted for. The numerous biomarker values available for each neonate over the follow-up are likely to be correlated. To account for this correlation, generalised estimating equations (GEEs) were used with exchangeable correlation (i.e. correlation between biomarker measurements within neonates is constant). GEEs assume linear relationships between each biomarker and time. However, the change in the biomarker values over time is not linear (there are peaks in biomarker values with gentamicin treatment which revert to baseline following cessation of treatment). These changes over time could be accounted for using polynomial equations (i.e. cubic terms) within the GEEs. However, with an increasing number of terms (i.e. knots) multi-colinearity is more likely, affecting the estimate of the association between each of the biomarkers and gentamicin treatment. Therefore, a better solution is to use regression splines, which fit the best-fitting fractional polynominal.

The mfp program in Stata provides a framework for carrying out both tasks simultaneously: selecting (fractional polynomial) functions of the biomarker with time, and the GEE of the biomarker with time. [19] Therefore we are able to account for the change in each of the biomarkers over time and the correlation between measurements. Accounting for both these aspects we are then left to explore the association between treatment and each of the biomarkers.

Postmenstrual age (weeks) was included in the models as it might have an impact on overall differences in biomarkers over the follow-up, and a term was included to assess if this impact changed with time (e.g. impact of gestation diminished/increased over time (later not reported)).

Actual mean difference and percentage difference in each biomarker with gentamicin treatment, as well as baseline values for each biomarker when not on treatment, were calculated. The treatment effects are adjusted for confounders to assess if other factors may explain the association between the biomarker and treatment. Potential confounders were identified from the following factors if they were significantly associated (p<0.05) with each of the biomarkers: the episode of treatment received by day of life, gestation (weeks), birth weight (kg), sex, Apgar score at 5 minutes, respiratory distress syndrome, received prophylactic indomethacin in the first week of life, received indomethacin (yes or no, for each day), received furosemide (yes or no, for each day), serum creatinine concentration by day of life, and occurrence of a co-morbidity (yes or no, for each day). Co-morbidities were any of the following: sepsis (blood culture positive), necrotising enterocolitis, cardiopulmonary resuscitation, need for oscillator/high flow oxygen ventilation/nitric oxide, need for inotropes, or surgery.

## Results

### Overview of Urine Collection and the Incidence of Gentamicin Therapy

A total of 41 neonates were recruited to the study. Summary characteristics are presented in Table 1. A median of 4 urine samples (Inter-quartile Range (IQR) 2–12) were collected for each neonate. The majority of urine samples measured between 1 and 5 ml, with the smallest amount being 200 µl in a few patients, but this was still adequate to perform all the assays. The median length of follow-up per neonate was 30 days (IQR 14–48). The first urine sample was taken at a median of day 7 (IQR 6–7).

**Table 1:** Baseline characteristics and clinical signs of neonates treated with gentamicin.

			Number				Mean biomarker value			
Gestation (weeks)	Number	Mean Birthweight (kg)	Males	Respiratory Distress	Indomethacin Prophylaxis	Acute Kidney Injury	Urinary KIM-1(ng/mg uCr)	Urinary NAG(IU/mg uCr)	Urinary NGAL(ng/mg uCr)	Serum Creatinine(micromol/l)
**<26**	6	0.758	3	6	6	4	3.64	0.28	1046.72	93.37
**26 to <28**	9	0.893	6	9	8	1	2.85	0.14	632.68	56.51
**28 to <30**	11	1.106	6	10	2	0	2.67	0.18	538.49	56.12
**30 to 32**	15	1.499	10	11	0	0	0.72	0.05	82.76	54.19

Patients are subdivided according to gestational age. Mean biomarker values presented include samples collected both on and off gentamicin treatment over the whole time course of inclusion in the study.

40 of the 41 patients (97.6%) received treatment with gentamicin during the first week of life. Twenty-one (51.2%) went on to have 2 or more treatment courses of gentamicin during the follow-up period. The median number of days of gentamicin treatment for any particular neonate was 5 (IQR 1–8) and median episodes of treatment was 2 (IQR 1–2). Only one participant did not receive any gentamicin. 8 patients were discharged home, 16 were transferred to other neonatal units, 2 were withdrawn from the study by their parents, 3 died, and 12 remained on our NICU at the end of the study follow-up.

### Evaluation of Novel Urinary Biomarkers in Cases of Confirmed AKI

**Figure 1:**
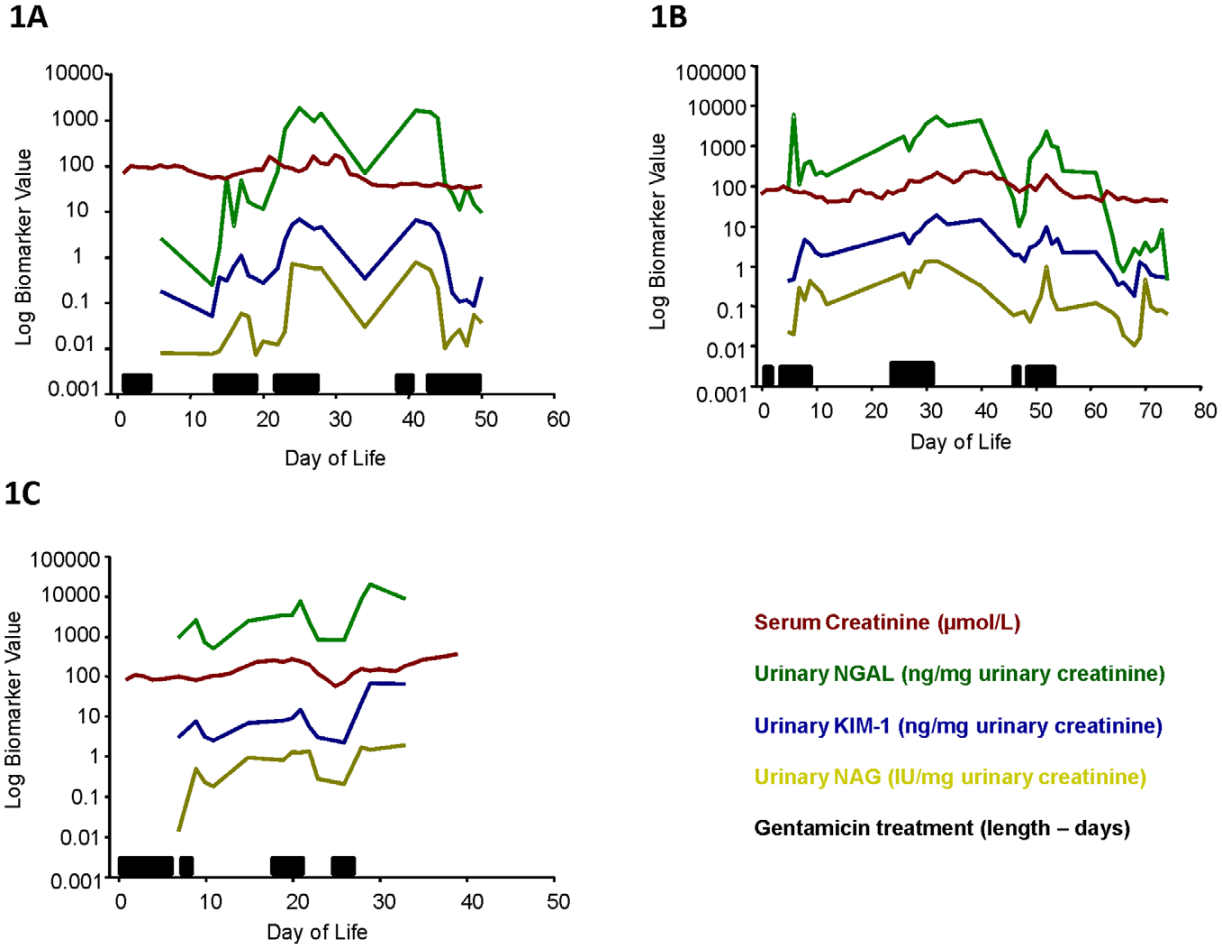
Longitudinal biomarker analysis of infants treated with multiple courses of gentamicin with a change in serum creatinine concentration (AKI). Representative figures demonstrating the longitudinal quantification of the biomarkers KIM-1 (blue; ng/mg. uCr), NGAL (green; ng/mg. uCr), NAG (yellow; IU/mg. uCr) and serum creatinine (red; µmol/L) for three infants treated with gentamicin (A–C). Gentamicin treatment episode and length of treatment (days) are indicated by the black horizontal bar on each figure for that individual patient.

Five patients developed AKI during the course of the study as defined by a sCr concentration >132.6 micromol/l (1.5 mg/dl) and/or oliguria (urine output <1 ml/kg/hour recorded at any point). In this patient group, we investigated the hypothesis that it was feasible to measure a panel of urinary biomarkers, and that in cases when serum creatinine was elevated, the urinary concentration of each of the novel mechanism-based biomarkers would also be increased. During episodes of AKI patients had significantly increased mean values of KIM-1 (mean difference 5.84 ng/mg uCr; 95% CI 3.77, 7.92), NGAL (mean difference 2031.7 ng/mg uCr; 95% CI 1351.4, 2711.9), and NAG (mean difference 0.53 IU/mg uCr; 95% CI 0.39, 0.68) compared to those without AKI. Representative figures are given for three infants (Fig. 1).

### Assessment of the Impact of Gentamicin Treatment on the Concentration of Urinary Biomarkers

**Figure 2:**
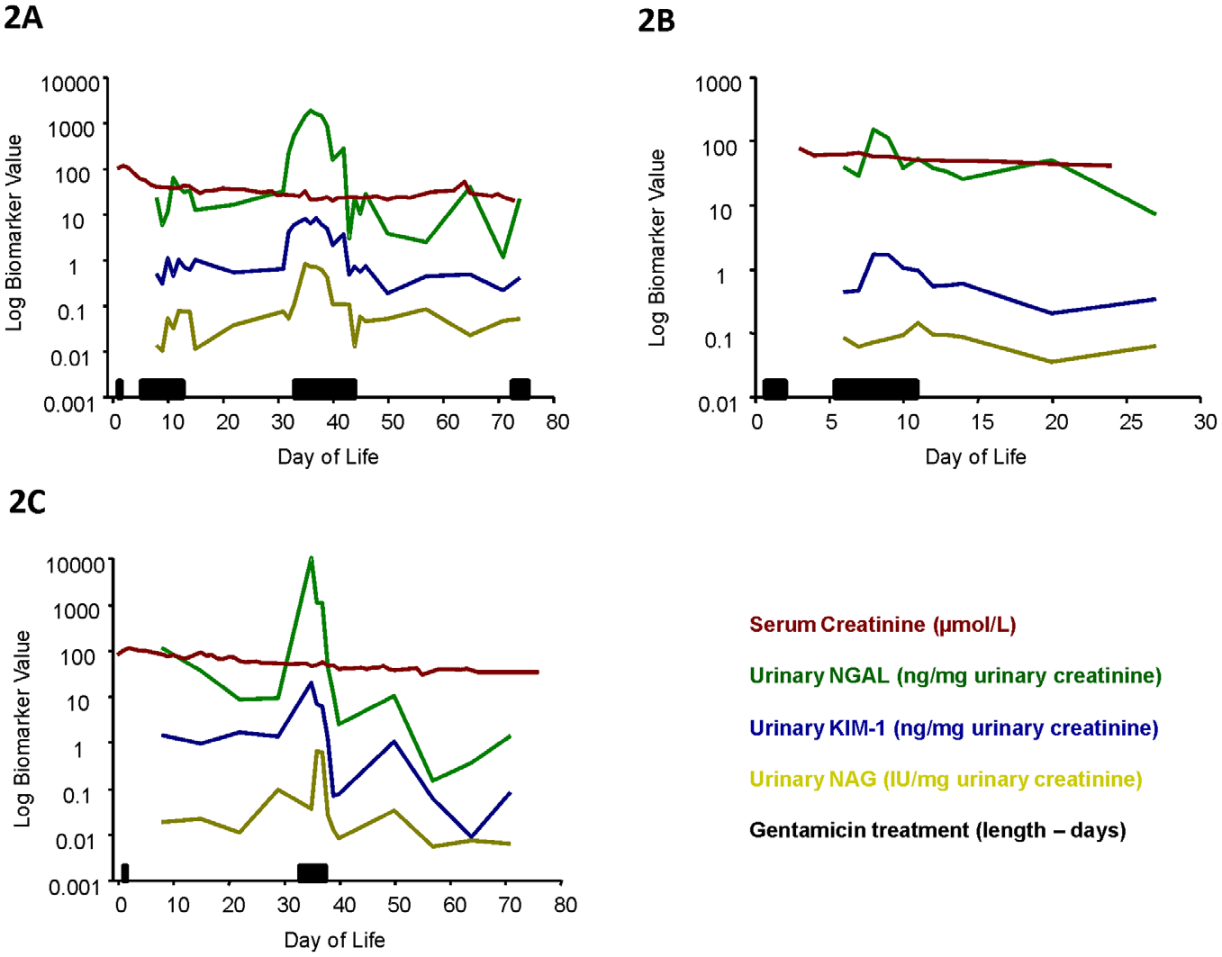
Longitudinal biomarker analysis of infants treated with multiple courses of gentamicin without a change in serum creatinine concentration. Representative figures demonstrating the longitudinal quantification of the biomarkers KIM-1 (blue; ng/mg. uCr), NGAL (green; ng/mg. uCr), NAG (yellow; IU/mg. uCr) and serum creatinine (red; µmol/L) for three infants treated with gentamicin (A–C). Gentamicin treatment episode and length of treatment (days) are indicated by the black horizontal bar on each figure for that individual patient.

After determining that measuring each of these urinary biomarkers was feasible in this patient group in the ICU setting using the method that we employed for urine collection, and that their concentration increased in the presence of confirmed AKI, we next explored the relationship between the urinary biomarker concentration and incidence of gentamicin treatment. Figure 2 illustrates representative data from three patients treated with multiple courses of gentamicin. Multiple treatment courses (21/41 patients), in particular, were associated with transient increases in KIM-1, NGAL and NAG. No elevation in any of the biomarkers investigated was seen in the one patient that was not exposed to gentamicin. Interestingly, in the patients without confirmed AKI that were treated with multiple course of gentamicin (16/21), the urinary concentration increase following gentamicin treatment was observed in the absence of a change in serum creatinine concentration. An analysis of the association between gentamicin treatment and biomarker values is presented in table 2 for all patients recruited to the study.

**Table 2:** Association between gentamicin treatment and the change in biomarker values.

		Effect on biomarker			
Predictors of variability of biomarker		KIM-1 (95% CI)(ng/mg uCr)	NAG (95% CI)(IU/mg uCr)	NGAL (95% CI)(ng/mg uCr)	Creatinine (95% CI)(µmol/l)
Mean difference in biomarker value from baseline whenreceiving gentamicin		1.64 (0.54, 2.75)	0.08 (0.02, 0.15)	453.6 (145.1, 762.2)	−4.64 (−8.64, −0.64)
**Predictors of variability of biomarker - adjusted for confounders**					
Mean difference in biomarker value from baseline whenreceiving gentamicin		1.35 (0.05, 2.65)	0.06 (−0.02, 0.13)	298.58 (−56.21, 653.38)	−8.6 (−13.1, −4.2)
Fixed effects: mean difference in biomarker over the follow-up					
Gestation: per week increase		0.22 (−0.44, 0.88)	0.01 (−0.03, 0.05)	33.17 (−145.36, 211.71)	−4.4 (−10.1, 1.2)
Received Indomethacin in the first week of life: compared to not		1.51 (−1.55, 4.57)	0.06 (−0.11, 0.23)	319.2 (−501.9, 1140.2)	−7.1 (−34.4, 20.2)
Time dependent fixed effects: mean difference in biomarker giventhe predictor					
Episodes of Gentamicin by a given day: per episode increase		–	−0.02 (−0.07, 0.02)	–	2.3 (−0.9, 5.5)
Co-morbidity on a given day: compared to not		1.06 (−0.71, 2.83)	0.12 (0.02, 0.22)	518.3 (30.4, 1006.2)	17.2 (11.3, 23.2)
Creatinine on a given day: per unit 1 increase		0.05 (0.02, 0.07)	0.004 (0.003, 0.005)	15.97 (10.02, 21.91)	–

The mean baseline biomarker values in the absence of any gentamicin treatment were 1.91 ng/mg uCr (95% CI 1.07, 2.76) for KIM-1, 0.13 IU/mg uCr (0.07, 0.19) for NAG, 425.4 ng/mg uCr (162.6, 688.3) for NGAL, and 62.39 µmol/l (53.1, 71.69) for creatinine.

### Determination of Baseline Urinary Biomarker Values and the Effect of Gentamicin Treatment

After observing the association between drug treatment and biomarker elevations, we next sought to establish baseline values for each patient to assess the impact and significance of changes in biomarker values. The mean baseline values, in the absence of any gentamicin treatment, were 1.91 ng/mg uCr (95% CI 1.07, 2.76) for KIM-1, 0.13 IU/mg uCr (0.07, 0.19) for NAG, 425.4 ng/mg uCr (162.6, 688.3) for NGAL, and 62.39 µmol/l (53.1, 71.69) for creatinine. There was a significant difference observed in the value of all three urinary biomarkers between those treated and those not treated with gentamicin on any given day (table 2). These figures take account of the variability and correlation in the biomarker measurements within neonates over the follow-up period, as described in the statistical analysis section. Between courses of gentamicin, each biomarker returned to a value not significantly different from the value in those patients not receiving gentamicin.

The factors associated with KIM-1 in the univariate analysis, and therefore treated as potential confounders in the multivariate analysis, included gestation, prophylactic indomethacin, co-morbidities and serum creatinine (sCr). The same factors were also associated with NGAL and NAG in the univariate analysis, with the addition of gentamicin episode number for NAG. The association between gentamicin treatment and KIM-1 elevations remained after adjusting for potential confounders (mean difference 1.35 ng/mg uCr; 95% CI 0.05, 2.65), but not for NAG and NGAL (table 2).

### Evaluation of the Impact of other Medications on Urinary Biomarker Elevations

The NSAID, indomethacin, is commonly prescribed to neonates in an ICU setting, the use of which has been reported to be associated with renal toxicity, although this would not selectively affect the proximal tubule. Indomethacin treatment had no significant effect on the urinary concentration of KIM-1 (−1.31 ng/mg uCr; 95% CI −6.06, 3.44) and NGAL (−543.15 ng/mg uCr; 95% CI −2119.88, 1033.58), or the urinary enzymatic activity of NAG (−0.09 IU/mg uCr; 95% CI −0.42, 0.24).

## Discussion

Animal models of paediatric gentamicin toxicity have suggested that immature rats (aged 10 days) developed gentamicin-induced nephrotoxicity earlier, and to a greater extent, than more mature rats. [5] The level of renal maturity in 10-day old rats is similar to that found prenatally in humans [5], suggesting that preterm neonates may be at increased risk of gentamicin-induced nephrotoxicity compared to older children. Thus, it is important to develop methods to investigate renal injury caused by drugs in this vulnerable patient group. Our proof-of-concept investigation shows that it is feasible to collect serial urine samples, and despite the fact that only small volumes of urine were collected, it was possible to measure a panel of urinary biomarkers in a reproducible manner.

We hypothesized that urinary concentrations of the novel urinary biomarkers, KIM-1, NGAL and NAG would be elevated during AG treatment in the absence of, or before, changes in serum creatinine. Within this study, we found significant elevations in three urinary biomarkers (KIM-1, NGAL and NAG) in premature neonates during courses of treatment with gentamicin, which occurred in the absence of a significant increase in sCr. Once gentamicin was withdrawn, the biomarkers returned to baseline values, similar to that found prior to AG treatment in the same infant. Those neonates who developed AKI had higher mean values of all 3 urinary biomarkers. Adjustment for potential confounders showed that the increase in biomarker value remained significant only for KIM-1, but the confidence intervals for NGAL and NAG bordered on significance which may reflect our small sample size. It is important to note that this investigation represents the first determination of the urinary abundance of KIM-1 following drug treatment in pre-term neonates. Our findings clearly need independent replication, but it is also important to ensure that future studies are adequately powered to determine whether one of the biomarkers has superior predictive ability compared to the others.

NGAL has previously been evaluated in neonates: in a study of 20 patients, although there was marked variability in urinary NGAL levels, it had a sensitivity of 31% for predicting oliguria, and a specificity of 90%. [20] Two more recent reports have provided reference values for urinary NGAL in premature neonates. [21], [22] A direct comparison with the values reported in our study, however, is not possible as different measurement methods have been used, and our values are corrected for urinary Cr whereas the other studies have reported only absolute values. Urinary NGAL serves as a biomarker of sepsis-induced kidney injury [23]; this is consistent with our finding that co-morbidities, including sepsis, were a significant confounder of the gentamicin-associated increase in urinary NGAL. This may indicate that changes in NGAL values are more closely associated with the septic state than with exposure to gentamicin, which would be consistent with its role in the regulation of inflammation. [24], [25] As with NGAL, the potential utility of NAG has previously been studied in neonates receiving AGs. Consistent with our findings, NAG increases during treatment with AGs and returned to baseline levels following drug withdrawal. [26] One recent study, however, found no significant difference in urinary NAG concentrations between neonatal infants treated with gentamicin for 10 days, and a control group. [27] NAG has also been shown to increase following perinatal asphyxia and the related renal ischaemic damage. [28].

There has been recent interest in measuring panels of urinary biomarkers in paediatric populations. In children attending an emergency centre, urinary NGAL and KIM-1 both demonstrated good accuracy in predicting patients with a greater than 50% reduction in estimated creatinine clearance. [29] These same two biomarkers were also useful as markers of worsening differential renal function in congenital obstructive nephropathy. [30] In preterm neonates, measurement of a panel of urinary biomarkers (which included NGAL and KIM-1) has recently been reported. [31] They found that values of these biomarkers were highest in the most premature. Our findings are in agreement with this, our baseline values were broadly similar, and we have accounted for gestational age in our analysis. The same group has also reported that urine biomarkers (again including NGAL and KIM-1) had good predictive value for AKI and mortality in very low birth weight infants. [32].

This study was designed to serve as a foundation for evaluation in future studies and demonstrates the feasibility of measuring a panel of biomarkers in urine samples from premature neonates. Our combination of regular weekly baseline measurements with an increased frequency of sampling during gentamicin treatment enabled us to assess changes in the measured biomarkers during these courses. Despite the relatively small number of neonates who participated in this study, we have been able to show significant changes in our biomarkers related to courses of gentamicin exposure via a longitudinal analysis. It is important to note that the development of a rapid urine dipstick test for KIM-1 [33] and bedside analysis for NGAL [34] suggests that these biomarkers could be measured in real time, increasing their potential clinical utility. In our study, we also noted that biomarker values decreased with increasing postmenstrual age. This suggests a differential sensitivity to renal insults with postmenstrual age, a concept reported pre-clinically [5] and is similar to our recent report of baseline levels of hepatic transaminases in preterm neonates. [35] However, this needs to be confirmed in a larger dataset.

Despite the novel findings of this current investigation, several limitations exist. First, the sample size was small. However, this was a proof-of-concept study, and the longitudinal analysis employed here provides a powerful insight into the dynamics and responses to drug treatment of each of the biomarkers investigated in the clinical setting. Due to the small number of confirmed cases of AKI, the relative sensitivity of each biomarker at reporting renal injury compared to currently used clinical standards could not be assessed with statistical confidence. However, the data provided within this investigation supports the feasibility of such future studies. Second, a number of neonates had only short stays in the NICU, often being transferred to regional units for continuation of their care closer to home. This meant that for some the period of follow-up following their baseline sample was relatively short. Further follow-up studies are planned to determine whether or not multiple courses of gentamicin exposure in neonates have a lasting effect on renal function. Third, 40 of our 41 participants received gentamicin in the first week of life, and a small number were on gentamicin when the first urine sample was collected. This means we do not have true, pre-gentamicin, baseline measurements of our biomarkers in all patients. Finally, although we have determined urinary biomarker changes associated with gentamicin treatment within this current investigation, we have not correlated this with clinical outcome or clinical decision making. It is important that future studies are appropriately designed so that guidance can be given on what clinical decisions (i.e. drug withdrawal or dose modification) need to be made in clinical settings when novel biomarkers are found to be elevated above baseline.

In conclusion, we have shown that it is possible to collect adequate volumes of serial urine samples to measure a panel of novel urinary biomarkers in premature neonates exposed to gentamicin. The application of the assays, the biomarkers investigated in this patient population in the ICU setting and the statistical analysis used, represent novel outcomes from this research. This proof-of-concept study will inform the design of further qualification studies of urinary biomarkers in larger neonatal cohorts which will include assessment of their relative sensitivity to predict AKI, compared to current clinical indicators. Furthermore, such studies should be carefully designed to answer the important questions as to whether changes in clinical management (stopping the drug or altering dosage) lead to better patient outcomes both in terms of treatment of their underlying infection, and also whether further more serious kidney injury is averted.
